# Riding the Wave of Ambivalence in Cell Biology

**DOI:** 10.3390/ijms25137348

**Published:** 2024-07-04

**Authors:** Sonia Emanuele, Michela Giuliano

**Affiliations:** 1 Department of Biomedicine, Neurosciences and Advanced Diagnostics (BIND), Biochemistry Building, University of Palermo, Via del Vespro 129, 90127 Palermo, Italy; 2 Department of Biological, Chemical and Pharmaceutical Sciences and Technologies (STEBICEF), Laboratory of Biochemistry, University of Palermo, Via del Vespro 129, 90127 Palermo, Italy

Increasing evidence clearly shows that most functional molecules in the cell exert a dual role depending on the specific interactive context, biochemical pathway, or subcellular localization.

Therefore, it is not surprising that a tumor suppressor may transit into an oncogene, an antioxidant may switch to a pro-oxidant, and a pro-survival factor can turn into a death promoting one. 

A huge number of examples may be provided to sustain this molecular ambivalence or even a more complex multifunctional capability exerted by different cellular factors. The ability to display different and sometimes opposing functions can be considered an evolutionary trick to make a limited number of factors exert diverse biological activities. This statement is perfectly fitting to the example of intrinsically disordered proteins (IDPs), a wide class of proteins with limited or extended disordered regions in their sequences, which confer conformational flexibility and adaptability to bind several interactors [1,2]. Intrinsic disorder is incredibly abundant in nature and highly associated with regulatory functions including signal transduction, gene expression, network assembly, and, more generally, the determination of cell fate. Intriguingly, disordered elements have also been found in well-known proteins with characterized functions, such as the transcription factor p53 [3], which can exert opposing roles in determining the cell fate, or the cyclin-dependent kinase inhibitors p21(waf1/cip1) [4] and p27(KIP1) [5], which are involved in cell cycle control.

Currently, the list of proteins with disordered elements has significantly increased, and this represents a key structural feature that may in part explain behavioral variability and pliability.

The story is even more complicated considering the plethora of posttranscriptional modifications that proteins undergo (including phosphorylation, acetylation, ubiquitylation, etc.) that modify their activity, as well as genetic or epigenetic regulation of their expression, which modify their cellular levels. Furthermore, conformational changes in domain or subunit that fold into more than one stable structure may occur, which are typical of the so-called “metamorphic proteins” [6]. Even enzymes, which for a long time have been considered more rigid proteins and typically well structured, when displaying a quaternary structure, may disassemble their subunits, change conformation, and reassemble the subunits, thereby changing their quaternary structure and functions [7].

In such an intricate landscape, we decided, as guest editors, to accept the journal’s proposal to promote the second edition of the Special Issue “Dual function molecules and processes in cell fate decision”, namely the 2.0 edition, that we describe here.

As mentioned above, p53 is one of the factors that displays ambivalent behavior since it can either canonically exert a tumor suppressor function or it can turn into an oncogene due to genetic mutations that promote tumor transformation and progression [8,9]. The first paper published in the Special Issue describes the interplay between oncogenic p53 and murine sarcoma viral oncogene homolog B (BRAF) in melanoma cells [10]. Notably, this contribution highlights the dual role of p53 in the response to a histone deacetylase (HDAC) inhibitor, ITF2357 (Givinostat), in *BRAF*-mutated melanoma cells. In particular, the authors provided evidence in this paper that wild-type p53 is able to sustain ITF2357-induced apoptosis, while oncogenic p53 exerts the opposite effect. The association of oncogenic p53 with oncogenic BRAF was found in the nucleus of melanoma cells and related to a lower tumor responsiveness to the effects of the compound compared to the wild-type p53 model. However, ITF2357 was capable of targeting both oncogenic p53 and BRAF as well as reducing their interaction. Overall, the HDAC inhibitor was considered a promising antitumor agent for melanoma-targeted therapy, and p53 status was a predictive factor for tumor responsiveness.

Another factor that can exert a dual role in the cell is the c-Jun-NH(2)-terminal kinase (JNK). This represents a stress kinase whose activation has been tightly associated with the production of reactive oxygen species (ROS) [11,12]. The role of ROS in the activation of the JNK pathway was clearly confirmed by antioxidants or ROS scavengers that could prevent the activation of this stress kinase [13]. The outcome of JNK activation is strongly dependent on the cellular context and the specific cell type. Although JNK has been widely documented to promote apoptosis, it was reported that the kinase may also serve an anti-apoptotic function [14]. The paper published by Varga et al. in this Special Issue [15] discusses the relationship between the JNK pathway and ferroptosis, a well-known type of cell death that has also been interpreted as a dual-function process. These authors show that a combination of JNK inhibitors with ferroptosis inducers enhances the cytotoxic effect in neuroblastoma RAS viral oncogene homologue (*NRAS*)- and Kirsten rat sarcoma virus (*KRAS*)-mutated tumor cell lines. In contrast, ferroptosis inhibitors counteracted the effect of the JNK inhibitors. Considering that the sole JNK-inhibitor treatment did not affect cell viability, the authors concluded that JNK inhibitors act by amplifying the effect of the ferroptosis inducers. This amplifying effect was not observed in other types of cell death promoted by oxidative stress, thus being specific to ferroptosis. The authors also provided evidence that intracellular glutathione (GSH) content/depletion represents an important candidate to modulate the anti-tumor effect of JNK inhibitors.

Intriguingly, ROS can also behave as dual-function mediators. It is widely known that the hormetic dose response of ROS can either promote pro-survival cell functions, including signal transduction regulation, when they are at low concentrations, or, overcoming a certain threshold, cause oxidative stress and consequent cell death [16,17]. Szarka et al. provided another contribution to this Special Issue in the form of a review, specifically focused on the dual function of ROS [18]. More precisely, they examined the relationship among three components: 1. an element: iron; 2. a process: ROS generation; and 3. a molecule: ascorbate, to show their dual functions and their role in cell fate decisions. Iron plays an important role as a component of many proteins involved in cell metabolism and detoxification [19]. However, the accumulation of free iron can trigger oxidative reactions in the presence of oxygen, resulting in ROS generation and oxidative stress [20]. ROS, in turn, at moderate concentrations, guarantee redox balance in the cell and are essential for different signaling pathways. On the other hand, an increase in their levels promotes cell injury and consequent cell death [16,17]. Ascorbate is a well-known antioxidant with a double face depending on its concentration and redox state. Its antioxidant power is exerted at low concentrations, at which it favors ROS elimination [21]. However, it turns into an ROS generator at high concentrations and in the presence of transition metals, including iron itself. The authors nicely describe this triad and critically discuss its involvement in the regulation of the cell survival/cell death balance.

Metabolic reprogramming is a hallmark of cancer, and recent strategies aim to target oncometabolites or modify tumor-specific metabolic pathways [22]. A nice paper by Lambrou et al. in this Special Issue examines the dual mechanisms of metabolism and gene expression induced by glucocorticoid treatment in leukemia cells [23]. Briefly, they provided evidence that different concentrations of prednisolone exerted distinct effects on glucose uptake and metabolism in tumor cells. According to their results, they hypothesized that high doses of prednisolone altered the membrane’s permeability and glucose uptake, or interfered with the glucose receptors, without exhibiting any cytotoxic effects. From a metabolic point of view, prednisolone was supposed to reverse the Warburg effect in leukemic cells and shift it towards autophagy. They found that tumor growth was not directly proportional to glucose consumption, analyzing different cell subpopulations: viable, necrotic, or apoptotic cells. They also performed metabolic and gene expression analyses, concluding that different types of leukemic cells exhibit different patterns of glucose metabolism.

Moreover, remaining on the subject of cancer, a very innovative contribution was provided by Morana et al. [24], who focused on the dual function of a well-known process, apoptotic cell death, highlighting the “*Apoptosis paradox in cancer*”. Apoptosis represents the most prominent type of programmed cell death with physiological relevance since it is implicated in maintaining tissue homeostasis, limiting cell population expansion, and eliminating damaged cells. Although for a long time, the loss of the ability to undergo apoptosis had been linked with tumor development and progression, more recently, a paradox has emerged as high-grade cancers have been found to be generally associated with high constitutive levels of apoptosis. One implication of this paradox is the apoptosis-mediated activation of cells of the immune system that promote a pro-oncogenic tumor microenvironment and produce evasion of cancer therapy. The authors presented an articulated overview of the implications of apoptosis in tumor biology, discussing the “double-edged” consequences of the process: on the one hand, its tumor suppressive nature through the elimination of malignant cells, while, on the other hand, the tumor promoter effects through stimulation of regenerative responses in the tumor microenvironment.

Finally, a contribution on developmental biology/differentiation was presented in a Special Issue by Hoffman et al., who provided a paper that describes how to obtain a mega-intestine with normal morphology by in silico modeling of postnatal intestinal growth in a *Cd97*-transgenic mouse [25]. Although not highlighting a specific duality in this process, the authors provide a nice model of cell fate decision. Indeed, they showed that changes in the autonomous specification of the intestinal cell fate induced by *Cd97* activation promote the formation of a mega-intestine with normal morphology.

Overall, the list of “*Dual function molecules and processes in cell fate decision*” is huge. To prolong the Special Issue would not be enough! We are glad that the two editions were successful and provided interesting and original contributions. We warmly thank all of the authors for the time dedicated to providing these excellent original papers and reviews. We also acknowledge the assistance of Lena Mao and the IJMS Editorial Office for their proposal to edit the second edition and for their efforts in the preparation of this Special Issue for publication.
